# Supplementary material for: Mechanisms governing the pioneering and redistribution capabilities of the non-classical pioneer PU.1
Source: Nat Commun. 2020 Jan 21;11:402. doi: 10.1038/s41467-019-13960-2 (PMC6972792; doi:10.1038/s41467-019-13960-2)
Supplement: Supplementary file 7 — Source data [file 41467_2019_13960_MOESM7_ESM.zip › Source_Data/FigureS2/FigureS2B_MotifScanOutput/KmeansCluster9/homerResults/motif7.similar.html]

motif7

## Information for motif7

C
T
A
G
T
C
A
G
A
T
G
C
C
G
T
A
C
A
T
G
A
C
T
G
C
T
G
A
G
C
T
A
T
C
A
G
A
C
G
T
C
T
A
G
C
T
G
A
  
Reverse Opposite:  

G
A
C
T
G
A
T
C
G
T
C
A
A
G
T
C
C
G
A
T
G
A
C
T
T
G
A
C
G
T
A
C
G
C
A
T
A
T
C
G
A
G
T
C
G
A
T
C
  

|  |  |
| --- | --- |
| p-value: | 1e-75 |
| log p-value: | -1.731e+02 |
| Information Content per bp: | 1.703 |
| Number of Target Sequences with motif | 209.0 |
| Percentage of Target Sequences with motif | 9.18% |
| Number of Background Sequences with motif | 796.3 |
| Percentage of Background Sequences with motif | 1.73% |
| Average Position of motif in Targets | 104.5 +/- 55.5bp |
| Average Position of motif in Background | 101.4 +/- 66.1bp |
| Strand Bias (log2 ratio + to - strand density) | 0.6 |
| Multiplicity (# of sites on avg that occur together) | 1.00 |
| Motif File: | file (matrix) reverse opposite |

### Similar de novo motifs found

|  |  |  |  |  |  |  |  |
| --- | --- | --- | --- | --- | --- | --- | --- |
| Rank | Match Score | Redundant Motif | P-value | log P-value | % of Targets | % of Background | Motif file |
| 1 | 0.777 | A G T C T C G A A G T C A G C T A C G T T A G C A G T C A G C T C A G T A T G C A G T C G A C T | 1e-58 | -134.435368 | 21.49% | 9.69% | motif file (matrix) |
| 2 | 0.819 | C G T A A C T G A T C G G A T C C G T A C T A G C A T G C T G A C T G A C T A G G A C T C T A G C A T G | 1e-34 | -80.389987 | 4.44% | 0.87% | motif file (matrix) |
